# A high resolution scanning electron microscopy analysis of intracranial thrombi embedded along the stent retrievers

**DOI:** 10.1038/s41598-022-11830-4

**Published:** 2022-05-16

**Authors:** Daniela Dumitriu LaGrange, Gianmarco Bernava, Philippe Reymond, Isabel Wanke, Maria Isabel Vargas, Paolo Machi, Karl-Olof Lövblad

**Affiliations:** 1 Neuroradiagnostic and Neurointerventional Division, Department of Radiology and Medical Informatics, Faculty of Medicine, University of Geneva, Geneva, Switzerland; 2 Division of Diagnostic and Interventional Neuroradiology, HUG Geneva University Hospitals, Geneva, Switzerland; 3 Division of Neuroradiology, Klinik Hirslanden, Zurich, Switzerland; 4 Swiss Neuroradiology Institute, Zurich, Switzerland; 5 Division of Neuroradiology, University of Essen, Essen, Germany

**Keywords:** Stroke, Biomedical engineering, Scanning electron microscopy

## Abstract

Endovascular treatment with stent retriever thrombectomy is a major advancement in the standard of care in acute ischemic stroke (AIS). The modalities through which thrombi embed along stent retriever following mechanical thrombectomy (MTB) have not yet been elucidated. Using scanning electron microscopy (SEM), we analyzed the appearance of thrombi retrieved by MTB from AIS patients, when embedded into the stent retriever. We observed that the organization and structural compactness vary for compositionally different thrombi. The modalities of attachment onto the stent vary according to thrombus composition and organization.

## Introduction

Acute ischemic stroke (AIS) is the first cause of acquired deficit and second cause of death in the occidental world. The standard of care in AIS was revolutionized by the introduction of endovascular treatment in clinical practice^1–9^. Mechanical thrombectomy (MTB) strategies allow for the cerebral blood flow to be restored by extracting the thrombus occluding the cerebral arteries. In particular, thrombectomy with stent retriever is highly effective and is used when thromboaspiration alone does not provide the necessary recanalization^10^. The modalities through which thrombi attach to stent retriever are still to be elucidated. Knowledge about the deformability and friction properties of main components of thrombi, red blood cells^11^ and fibrin^12,13^, enables scientists to design parametric studies in vascular phantoms and to anticipate the effectiveness of various thrombectomy techniques^14,15^. Nevertheless, compared to thrombi artificially generated in vitro, thrombi retrieved from patients are inherently more complex and diverse in terms of composition, morphology and mechanical properties^16,17^. A recent study, focused on mechanical properties of human stroke thrombi retrieved with endovascular techniques, reports that thrombi with increased fibrin/platelet content display an increase in stiffness, compared with thrombi rich in red blood cells^17^. The latter are also known to be more vulnerable to extraction with MTB^18^. Although visualization of how patient thrombi embed in the stent retriever as a result of thrombectomy can provide useful information, it remains an underexplored topic. Among the ex vivo characterization techniques, scanning electron microscopy (SEM) can render morphological information about the thrombus cellular content and fibrin organization^19,20^ and is the only technique capable of providing with high resolution relevant details for the thrombus structure in relationship to the attachment onto the stent. In this respect, the ex vivo studies conducted so far, which are scarce and limited to the outer view of thrombi, point out mechanical entrapment and adhesion as main means of thrombus incorporation^21,22^.

In this study, we analyzed with microscopy images the modalities in which compositionally different thrombi are incorporated into the stent, when retrieved form patients suffering from AIS, and we highlighted their underlying structural characteristics, their commonalities, and their differences when anchoring on to the stent.

## Results

Table 1 highlights the main characteristics of thrombi analyzed in this study, together with the following information: the arterial occlusion site from where each thrombus was retrieved, the type of stent retriever used for retrieval, and the recanalization outcome. As the examined thrombi vary in composition, we classified them as: red blood cells rich thrombi, with an estimated volumetric content of red blood cells (RBCs) > 95%, fibrin rich thrombus, with an estimated volumetric content of RBCs < 5%, and intermediate thrombi, having an intermediate composition between RBCs rich and fibrin rich. The RBCs rich thrombi have typically an RBCs rich core with various degrees of compactness, surrounded by an outer layer of fibrin. The intermediate thrombi are having a compact structure, formed of fibrin and platelets, in which various sizes of RBCs aggregates are encased. The fibrin rich thrombus analyzed in our study has a sheet like morphology. Through examination with SEM, we identified several commonalities and differences among the different modes in which thrombi embed along the stent retrievers. All the thrombi we examined appear to be wrapped, within some extent, around the stent struts. However, in case of RBCs rich thrombi, the stent struts appear to be protruding through the thrombus matter, a feature that we found missing in case of intermediate and fibrin rich thrombi. We also found that the RBCs rich or intermediate thrombi are making close contact with stent struts, at sites where they conform with the strut shape. This behavior indicates an affinity between thrombus and stent surface, and thrombus ability to deform, by wetting the stent. Although not a frequent mode of becoming embedded into the stent, the thrombus can also be found trapped between the adjacent stent struts. An feature which we observed, for some of the stents incorporating thrombi, was the existence of bridges composed of fibrin, with or without cellular material, between proximal stent struts. We also observed at the surface of some thrombi irregular patches, or thinly shaped foils, which we regard as vascular tissue removed during thrombectomy. We illustrate our findings with relevant examples below.

**Table 1:** Characteristics of retrieved thrombi.

Case	Occlusion location*	Stent retriever**	TICI score***	Type of thrombus****	Observed type of contact*****					Indications of vascular damage******	Distal embolization*******
					Protrusion	Wetting	Trapped	Wrapping	Bridges		
1	M2	Trevo 4 × 20 mm	3	RBCs rich	V	–	–	V	V	–	–
2	M2	Trevo 3 × 20 mm	3	RBCs rich	V	V	–	V	V	V	–
3	M2	Trevo 3 × 20 mm	3	Intermediate	–	V	–	V	–	–	M4
4	M1	Solitaire 4 × 40 mm	3	Intermediate	–	V	–	V	–	V	–
5	M1	Solitaire 4 × 20 mm	2c	Intermediate	–	V	–	V	–	V	M3
6	M1	Trevo 4 × 30 mm	3	Intermediate	–	–	–	V	–	V	–
7	M1, M2	Trevo 4 × 30 mm	3	Intermediate	–	V	V	V	V	–	–
8	M2	Catchmini 3 × 15 mm	3	Fibrin rich	–	–	–	V	–	–	–

*Thrombi were situated within segments of middle cerebral artery (MCA), for all the investigated cases, and retrieved with one pass MTB.

**TREVO Striker, Kalamazoo, Michigan, USA; Solitaire Medtronic, Minneapolis, USA; Catch-Mini, Balt, Montmorency, France.

***According to Thrombolysis in Cerebral Infarction (TICI) scale, used for grading the result of recanalization therapy.

****RBCs rich: red blood cells rich core wrapped in a thin outer layer of fibrin. Intermediate: regions of red blood cells agglomerates are relatively scarce and encased in large regions of compact fibrin and platelets. Fibrin rich: thrombus composed mainly of fibrin, with overall sheet like aspect.

*****Protrusion: stent struts are penetrating through segments of thrombus. Wetting: Thrombus conforms with the surface of the stent strut. Trapped: thrombus trapped between adjacent stent struts. Wrapping: thrombus material is wrapped around stent struts. Bridges: fibrin, interlaced with various amounts of cellular material, in the form of a film anchored on adjacent stent struts.

******Fragments of sheet like structures at thrombus surface, indicative for possible vascular tissue removal during thrombectomy.

*******Vascular occlusion at a distal MCA segment following thrombus fragmentation and migration of thrombus fragments.

### Red blood cells rich thrombus

**Figure 1:**
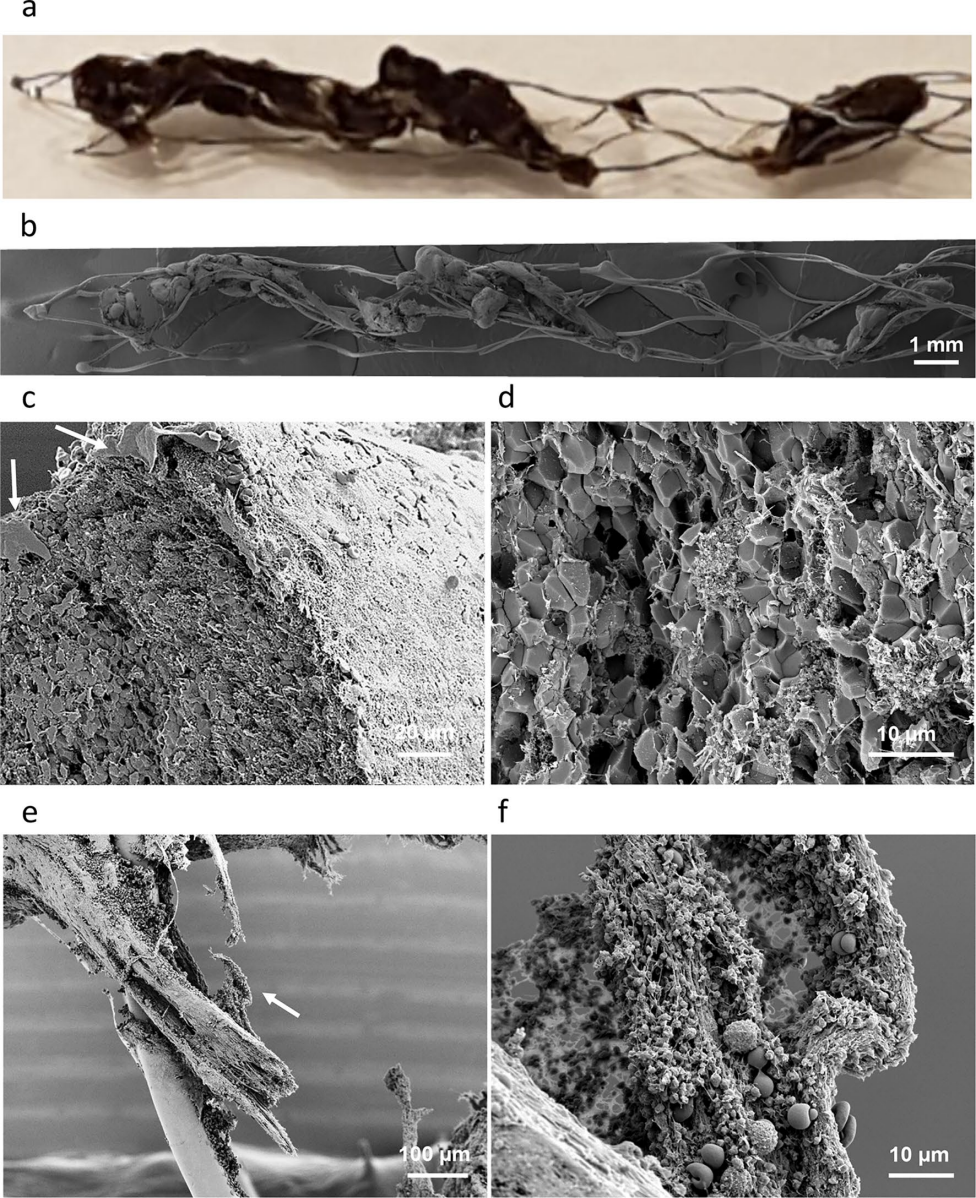
RBCs rich thrombus incorporated onto the stent retriever (Case 2 in Table 1). (**a**) Optical micrograph. (**b**) Collage of SEM micrographs. (**c**) Cross section of a thrombus segment, showing a compact core, porous periphery and fibrin outer layer. Remnants of vascular tissue are visible at the thrombus surface (arrows). (**d**) High magnification view of the compact core composed of polyhedrocites. (**e**) Fibrin strings are found in between thrombus segments. (**f**) White blood cells and platelets are attached to the fibrin strings (higher magnification view of (**e**), region marked with arrow).

**Figure 2:**
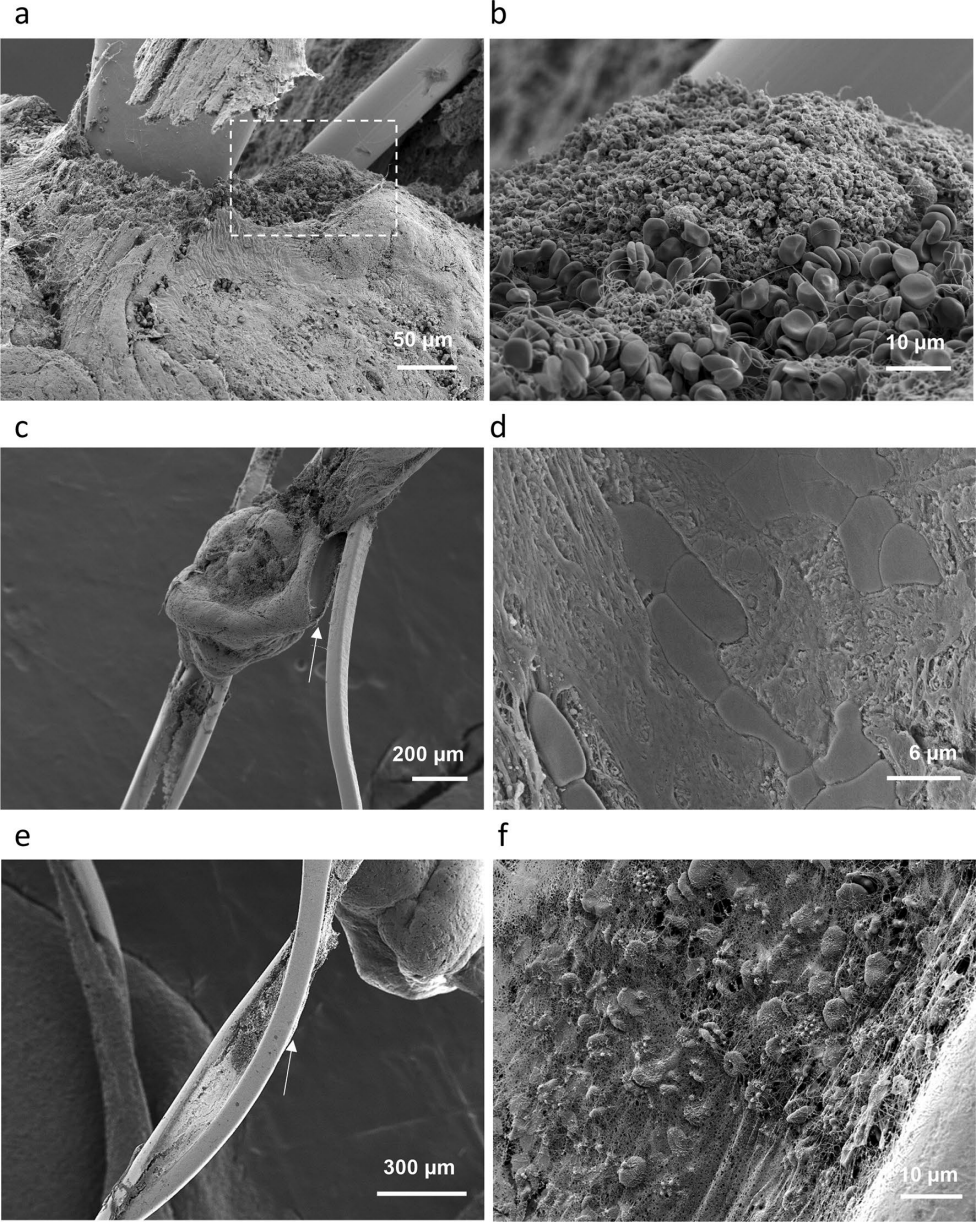
Modalities of RBCs rich thrombus attachment onto the stent. (**a**) Stent strut protruding through thrombus. (**b**) Higher magnification view from (**a**) (dashed rectangle), showing a platelets cap and biconcave RBCs. (**c**) Thrombus conforming with the stent strut. (**d**) Higher magnification view from **c** (region indicated by arrow), illustrating the thrombus contact area with the stent. (**e**) Bridges of fibrin between the adjacent stent struts. (**f**) High magnification view from (**e**) (region indicated by arrow).

Figures 1 and 2 are examples illustrating an RBCs rich thrombus incorporated into the stent. As thrombus spirals along the stent, its segments are anchored on single or multiple stent struts at a time—Fig. 1a,b. The RBCs rich thrombus segments display a compact core of polyhedrally shaped red blood cells and an outer layer formed of fibrin, characteristic for cerebral arterial thrombi^23,24^—Fig. 1c,d and Supplementary Fig. S1. The polyhedral shape of red blood cells is acquired due to compressive forces in vivo, during thrombus formation, and is considered a marker for intravital thrombus contraction^25–27^. The porosity of thrombus increases towards its periphery where platelets and white blood cells are also present along with fibrin. The segments of thrombus are interlinked by fibrin strings—Fig. 1e,f. The various modalities through which thrombus is incorporated into the stent are illustrated in Fig. 2. Protrusion of the stent through the thrombus (Fig. 2a,b; also Supplementary Fig. S2) occurs at sites with loose cellular packing, where the red blood cells are biconcave in shape. The thrombus can deform, wetting the stent surface—Fig. 2c,d. We also observed, between the double struts of the stent mesh, the existence of films, or bridges, of fibrin, with or without cellular content—Fig. 2e,f. It is unlikely that such fibrin bridges are native to the original thrombus that caused the stroke. Most probably, the bridges across the stent struts are formed during the retrieval process, and they potentially aid in securing the thrombus attachment.

### Intermediate, compact thrombus

**Figure 3:**
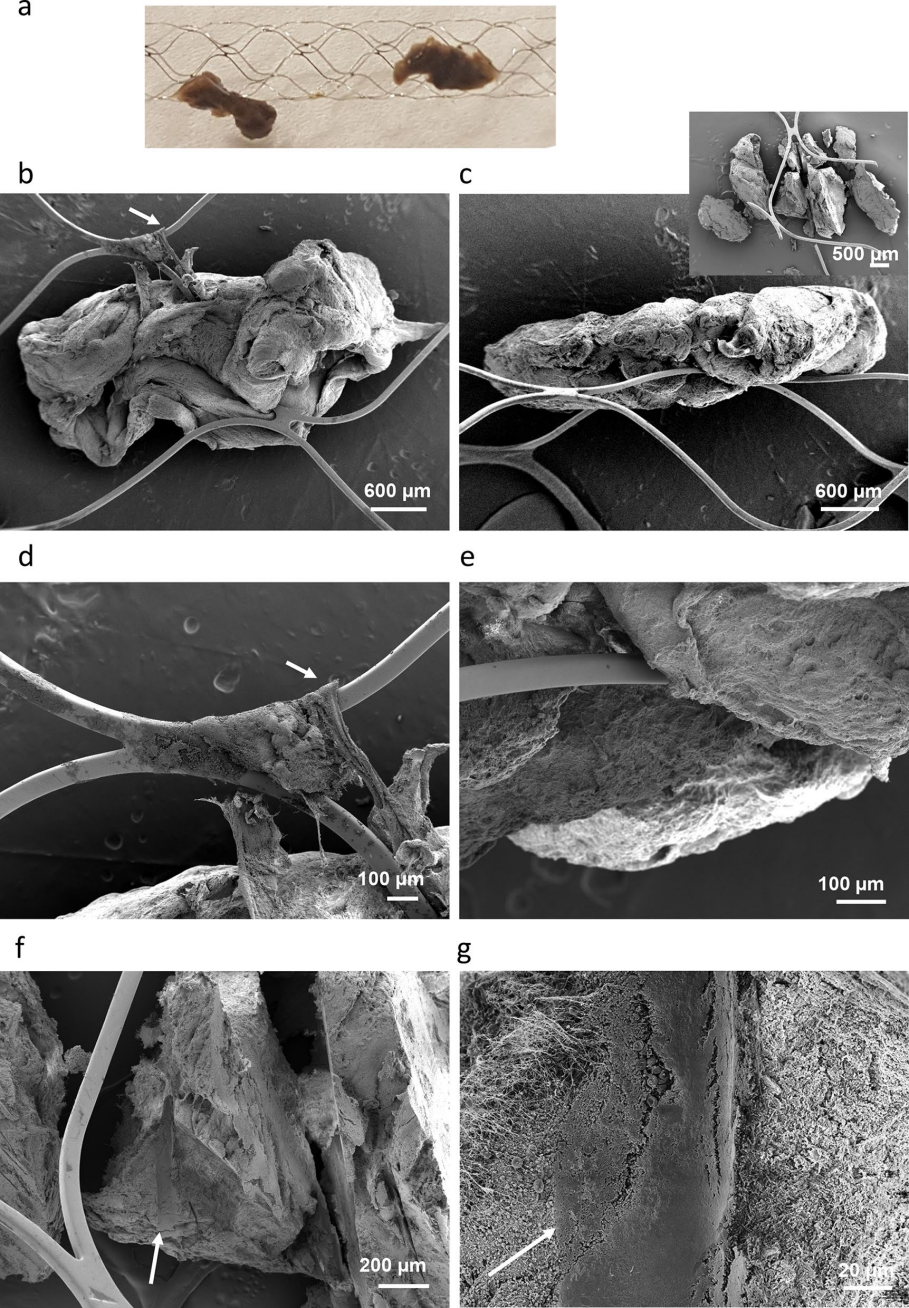
Intermediate thrombus incorporated into the stent retriever (Case 4 in Table 1). (**a**) Optical micrograph. (**b**, **c**) SEM view of thrombus on stent. Insert in (**c**) View of sectioned thrombus and the stent struts at the anchoring site. (**d**) Fibrin strings wetting the stent surface (indicated by arrows in **a**, **d**). (**e**) Thrombus clutches onto the stent struts. (**f**) Section through the compact thrombus, showing the contact region with the stent strut (indicated by arrow). (**g**) Higher magnification view of the thrombus surface at the contact with the stent (indicated by arrow).

**Figure 4:**
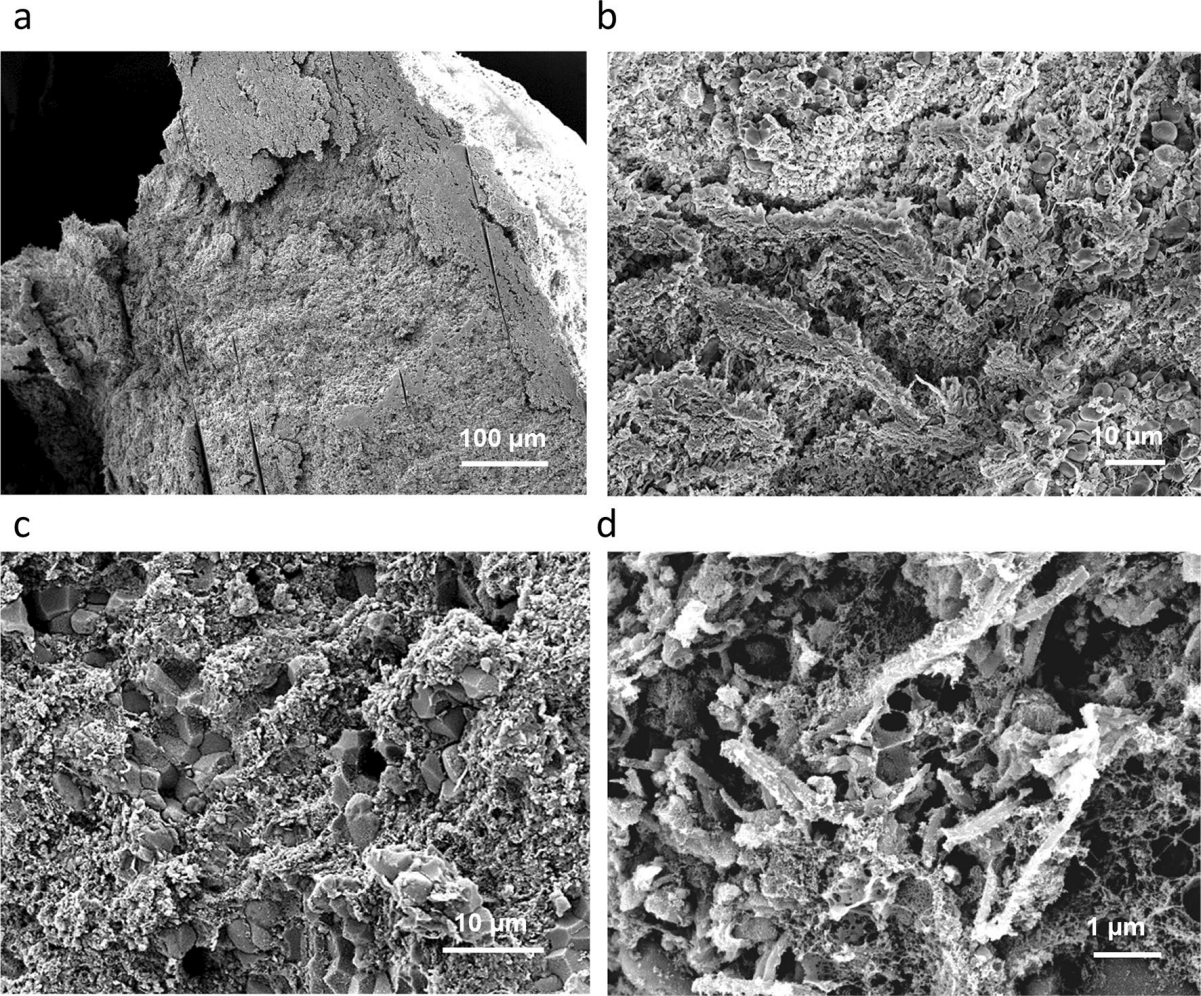
Compact structure of an intermediate thrombus. (**a**) Cross section of thrombus in SEM view. (**b**) Compact structure of thrombus periphery, with clusters of RBCs encased in a compact matrix of platelets and fibrin. (**c**) Compact core of thrombus, showing aggregates of polyhedrocites encased in a compact matrix of fibrin and platelets. (**d**) Higher magnification view from (**c**), showing the fibrin-platelets matrix.

When embedded into the stent, intermediate thrombi are wrapped all around the struts and, in some cases, they clutch the struts. Illustrative examples are presented in Fig. 3 and in Supplementary Fig. S3. The compact organization of intermediate thrombi is illustrated in Fig. 4 and Supplementary Fig. S4. Intermediate thrombi can wet the surface of the stent, either at sites where peripheral fibrin is organized in parallel strings (Fig. 3d; Supplementary Fig. S3), with an overall flexibility and affinity for the stent surface, or when compact thrombus deforms and clutches the stent. Remnants of vascular tissue, found at the surface of retrieved thrombi, are illustrated in Supplementary Fig. S5. Supplementary Fig. S6 presents an intermediate thrombus trapped between the stent struts.

### Fibrin rich thrombus with sheet like morphology

**Figure 5:**
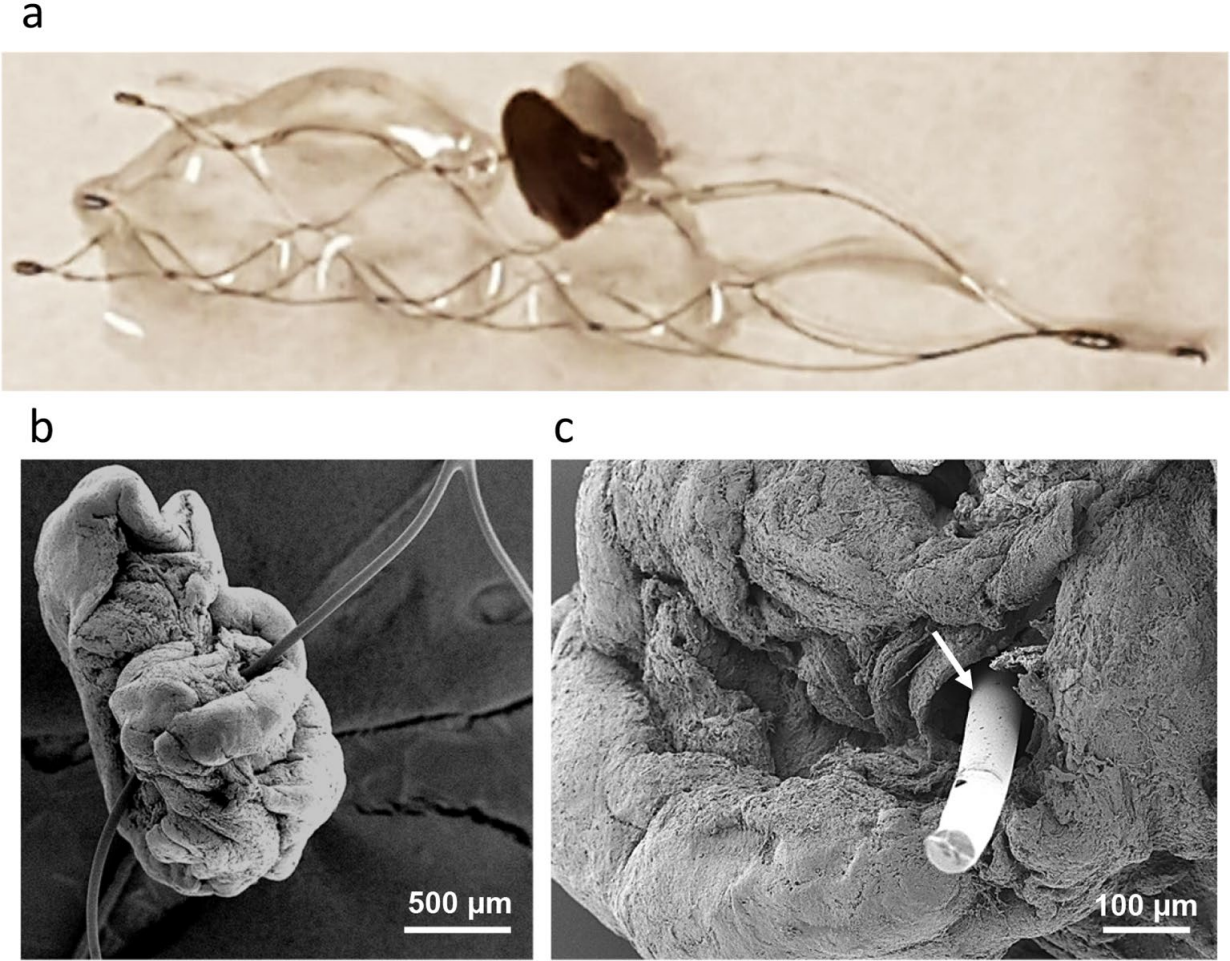
The fibrin rich thrombus integrated in the stent retriever (Case 8 in Table 1). (**a**) Optical micrograph. (**b**) Low magnification SEM view. (**c**) Closer SEM view at the thrombus-stent interface (the stent strut was cut, to allow better viewing). There is no adhesion between the thrombus and the stent strut (arrow).

The fibrin rich thrombus is integrated in the stent by incorporating one of the stent struts—Fig. 5. The thrombus has a non-adhesive behavior and displays a dense, fibrin rich, outer surface (Fig. 5c).

**Figure 6:**
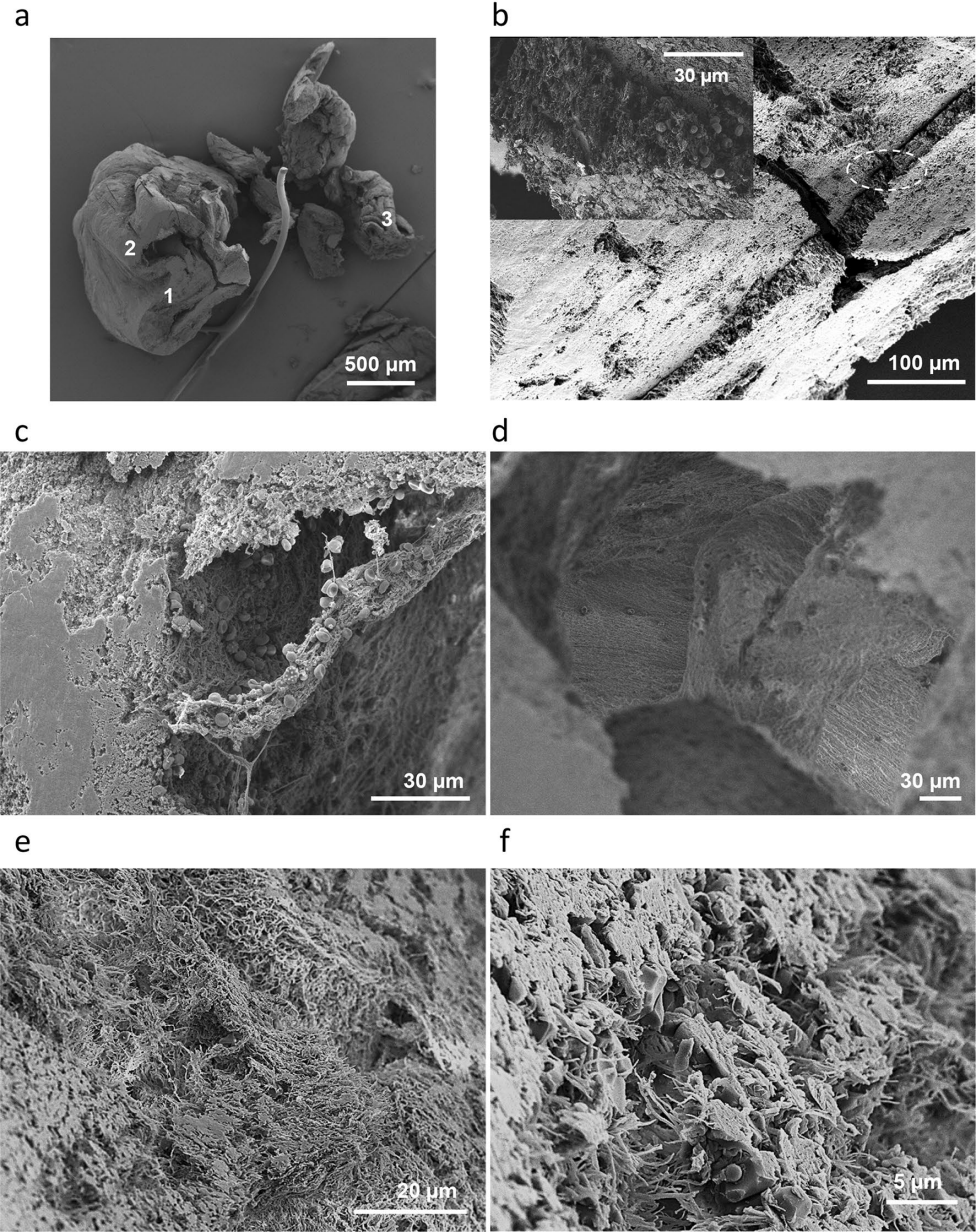
Analysis of fibrin rich thrombus. (**a**) Cross section of the thrombus. (**b**) Closer view of the fracture visible on the bigger section of thrombus (marked with “1” in **a**), and in inset higher magnification of the area marked by the dashed oval. (**c**), (**d**) Closer view of the region marked with “2” in (**a**). (**c**) Cross section into the wall and scattered red blood cells and white cells inside the cavity. (**d**) Broad view inside the cavity. (**e**, **f**) Cross sections of thrombus segments (marked “3” in **a**) that were wrapped around the stent strut. (**e**) Loosely packed fibrin region. (**f**) Compact region with polyhedral red blood cells, white cells in between the fibrin.

Figure 6a depicts fragments of the same thrombus after being sectioned along perpendicular directions. The fibrin is the main and ubiquitous structural element, while red blood cells content is scarce and non-uniformly distributed. The larger thrombus section reveals folding of a dense fibrin sheet (100–200 µm), with fibrin fibers being visible in higher magnification micrographs. The cross section indicates that the fibrin fibers are organized in bundles with a preferred parallel orientation to each other and perpendicular to the stent direction (Fig. 6a,b). White cells are incorporated into the dense fibrin sheet, with an estimate density of 2-to-4 cells per 1000 µm^3^, or roughly 15% of the volume, while the fibrin fibers occupy an estimated 70–80% of the local volume. The folding of fibrin sheet forms a cavity, which was inconspicuous prior to sectioning. Infrequent clusters of biconcave red blood cells and white blood cells are scattered through the cavity (Fig. 6c). A closer view within the cavity and examination of the inner walls also shows that fibrin fibers aggregate in large bundles which are cross linked and aligned in parallel to each other (Fig. 6d). After fragmenting the thrombus, we had a closer look at the part of thrombus originally wrapped around the stent strut—the smaller fragments in Fig. 6a. We observed that loosely packed microregions of fibrin with no cellular content (Fig. 6e) alternate with microregions of fibrin mesh encasing polyhedral red blood cells and white cells (Fig. 6f). The estimated cellular content, based on microscopy images in the latter case, is 10–20% of the local volume. Most importantly, in the region of thrombus wrapped around the stent strut the porosity is considerably higher, compared with the part distant from stent.

We summarize our findings for the fibrin rich thrombus as follows. The fibrin rich thrombus displays a sheet like morphology with two distinct types of structural organization. One, which constitutes most of thrombus volume, is compact and textured, as it consists in fibrin bundles connected to each other and uniformly aligned, in aggregates with high aspect ratio (100–200 µm thick, several hundreds of microns wide). In the direction of fibrin bundles, the bending angle of thrombus sheet is large, which suggests resistance to deformation. In other directions than along the fibrin bundles, the fibrin sheet proves more flexible, as undergoes bending at smaller angles and torsional deformation. The other type of structural organization consists in porous and randomly oriented micro-regions, tens of micrometers in size, of fibrin, with and without cellular content. The overall porosity, the randomness, and the size of the heterogeneous microregions allow deformation in multiple directions and thrombus wrapping around the stent strut.

## Discussion

The RBCs rich thrombus incorporates multiple segments which at the core are compact and consist in aggregates of red blood cells shaped as polyhedrocites. The compact aggregates evade anchoring onto the stent. In contrast, peripheral to the compact core and interconnecting thrombus segments, there are loosely packed volume regions of biconcave red blood cells and fibrin. The loosely packed regions of RBCs rich thrombus are the parts engaged in attachment onto the stent struts: they allow the struts to pass through, they are deformable and show surface affinity for the stent material. The intermediate thrombi, although they are compact, can deform. In this case, the extent of grain boundaries between thrombus components, the RBCs aggregates and the fine fibrin/platelets matrix, can be a contributor to thrombus behavior. The outer layers of fibrin strings can aid, in case of intermediate thrombi, the adhesive attachment to the stent. The incorporation of the fibrin sheet thrombus into the stent is non-adhesive and relies on foldability, or in other words on the ability of fibrin to wrap around the stent strut.

We show that variations in composition and compactness accompany the modalities of thrombus attachment onto the stent. The polyhedrocites core in RBCs rich thrombi, which is also known as a marker for intravital contraction, is of particular importance for response to treatment in AIS. When present, compact red blood cells regions make thrombi less susceptible to external fibrinolysis^28^. In addition, from the point of view of thrombus mechanical properties, intravital contraction is a contributor to stiffness^17^. Our study hints that the polyhedrocites core does not engage in thrombus attachment onto the stent. We found that RBCs rich thrombi embed onto the account of non-compact regions. Intermediate compositions, in terms of RBCs and fibrin content, lead to overall more compact but also deformable thrombi. Nevertheless, regardless of thrombus composition, the extent of non-compact volume regions, the ability to deform, and the affinity for the stent surface are the features that favor thrombus embedding into the stent.

The advantages of endovascular treatment are beyond doubt, as MTB improves recanalization rates in AIS patients and decreases associated morbidity and mortality^29,30^. It is becoming recognized that the use of stent retrievers is associated with vascular injury^31–34^. It is therefore not surprising to find in our study remnants of vascular tissue attached to the extracted thrombi.

A limitation of our study is the heterogeneous collection of stent retrievers used for MTB. This is partially due to the limited access to retrieved thrombi shortly after MTB, and limited possibilities in selecting samples according to the stent type. Most of the retrieved thrombi detach from the stent retriever shortly after intervention. It is also recognized that thrombi undergo structural changes, during and after retrieval process with MTB^35^. Also, thrombi are sometimes only partially removed from the occluded arteries. Even within these limitations, examining how thrombi embed along the stent retrievers offers an understanding of the underlying structural features which are responsible for thrombus capture into the stent. Standard procedures are needed, indeed, as such that the results of structural analysis of thrombi retrieved with MTB can be correlated with the structural organization of thrombi at the arterial occlusion site, in AIS patients. So far, and with the purpose of recognizing clinical neuroimaging signs predictive for the treatment outcome^36,37^ an empirical dichotomous categorization was established for the underlying histopathology of thrombi. According to this categorization, thrombi with a fairly rich content in RBCs are vulnerable to mechanical thrombectomy (MTB)^38,39^ and fibrin rich thrombi are resilient to endovascular techniques^39^. However, for enabling personalized treatment, innovative approaches should link clinical neuroimaging with the biomechanical factors governing thrombi incorporation into the stent retriever, and nevertheless with thrombi structural organization. Microstructural examination of human thrombi can help, in perspective, fill the existing knowledge gaps.

## Conclusions

The incorporation of thrombi along the stent retrievers engages thrombus regions that are deformable on the account of enhanced porosity or adequate composition. The capture can be adhesive, when thrombus is wetting the stent, and non-adhesive, when thrombus folds around the stent struts without making a close contact. In RBCs rich thrombi, stent struts can protrude through non-compact volume regions. The extent of compact and non-compact regions in intracranial thrombi, the composition of thrombus, and the adhesive affinity for the stent surface are important features for thrombus capture into the stent.

## Methods

The followed procedures were in accordance with the ethical standards of the responsible committee on human experimentation (institutional and national) and with the *Helsinki Declaration* of 1975, as revised in 2008. The study was approved, under the project ID 2018-00476, by the Cantonal Commission of Research Ethics (Commission cantonale d’éthique de la recherche, CCER) Geneva, who waived the requirement for informed consent, under the Art.34 LRH, Art.37-40 ORH.

### Endovascular technique

The thrombi were retrieved from patients suspected of suffering an acute ischemic stroke (AIS), who received whole brain stroke CT protocol^5^ and were referred for MTB intervention. Thrombectomy procedures were performed under general anesthesia using a bi-plane C-arm (Allura Clarity FD20, Philips Healthcare, Best, the Netherlands) via a common femoral artery approach. A guiding catheter was placed in the concerned artery at neck level. A large-bore aspiration-catheter was advanced over a microcatheter and a microwire up to the thrombus, while the microcatheter was advanced beyond the thrombus.

Subsequently, a stent retriever (Table 1) was advanced through the microcatheter and unsheathed across the thrombus. Hence, the stent retriever was gently retrieved inside the large bore aspiration catheter while a negative pressure was applied through it by using a vacuum system.

### Sample preparation and microscopy technique

Upon retrieval, thrombi integrated onto the stent were first immersed in formalin (4%), and immediately after were transferred in glutaraldehyde (2.5%), where they were kept overnight at 4 °C. Subsequently, samples were washed in phosphate buffer solution (PBS) 10X three times for 20 min each, dehydrated in solutions of ascending concentrations of ethanol (50, 60, 70, 80, 90, 100%) for 15 min each time, and dried using critical point drying. The samples were mounted on SEM stubs using carbon tape and carbon paint and sputtered with a 5 nm AuPd (80%/20%) coating. The microscopy observations were performed with an ultra-high-resolution field-emission Zeiss Merlin SEM, equipped with SmartSEM 6.06 service pack 6 software and a Gemini II column, using the Everhart–Thornley secondary electron detector, 5 kV acceleration voltage and 500 pA probe current.

## Supplementary Information

Supplementary Information.

## Data Availability

The data that support the findings of this study are available on request from the corresponding author.
