# Supplementary material for: A high resolution scanning electron microscopy analysis of intracranial thrombi embedded along the stent retrievers
Source: Sci Rep. 2022 May 16;12:8027. doi: 10.1038/s41598-022-11830-4 (PMC9110407; doi:10.1038/s41598-022-11830-4)
Supplement: Supplementary file 1 — Supplementary Information. [file 41598_2022_11830_MOESM1_ESM.pdf]

A high resolution scanning electron microscopy analysis of intracranial thrombi embedded along the stent retrievers

Daniela Dumitriu La Grange [1]*, Gianmarco Bernava [2], Philippe Reymond [1], Isabel Wanke [3 4 5], Maria Isabel Vargas [1 2], Paolo Machi [1 2], Karl-Olof Lövblad [1 2]

[1] Neuroradiagnostic and Neurointerventional Division, Department of Radiology and Medical Informatics, Faculty of Medicine, University of Geneva, Geneva, Switzerland

[2] Division of Diagnostic and Interventional Neuroradiology, HUG Geneva University Hospitals, Geneva, Switzerland

[3] Division of Neuroradiology, Klinik Hirslanden Zurich, Switzerland

[4] Swiss Neuroradiology Institute, Zurich, Switzerland

[5] Division of Neuroradiology, University of Essen, Essen, Germany

* corresponding author, e-mail: Daniela.DumitriuLagrange@unige.ch

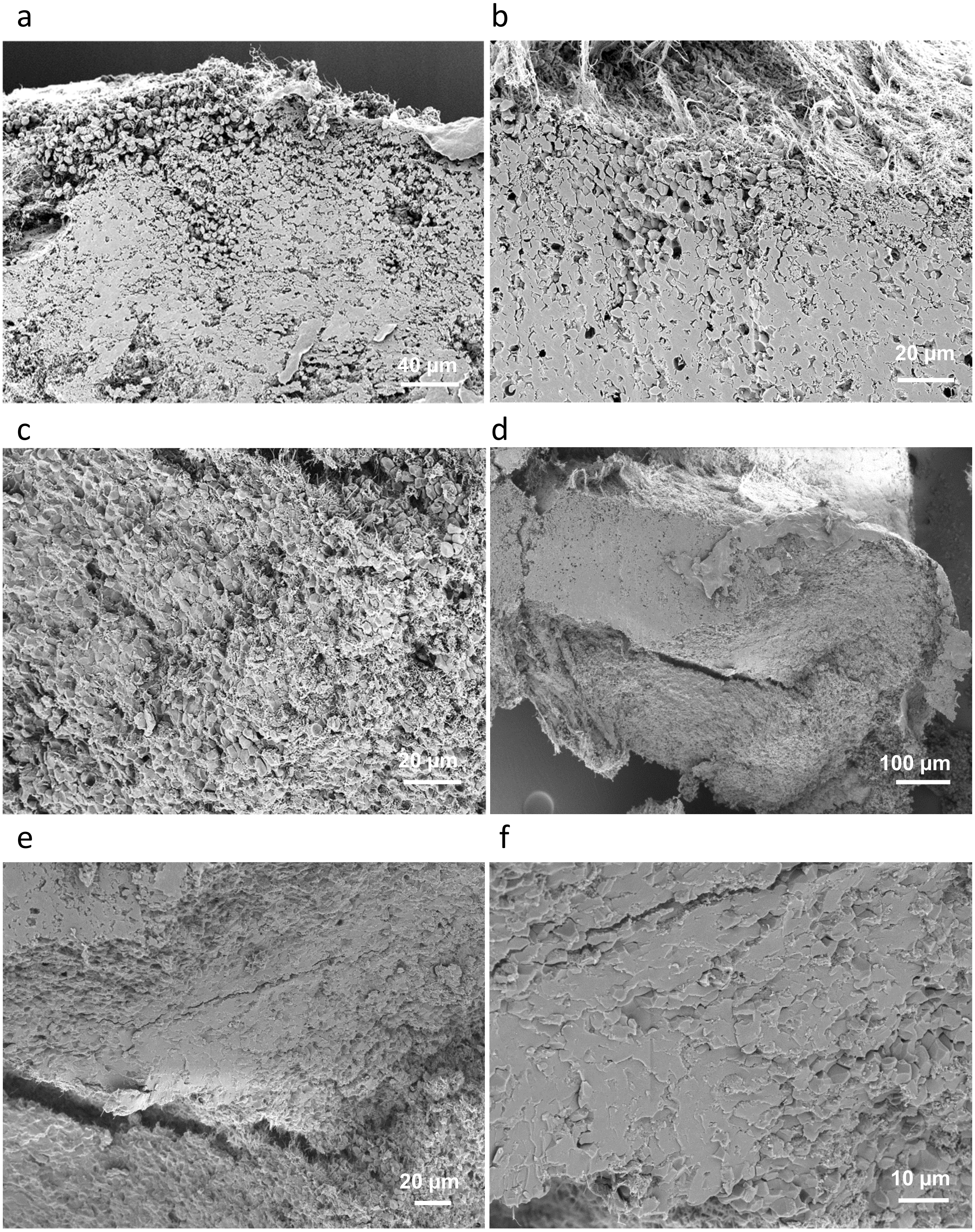

Supplementary Fig. S1 : Cross sections through the RBCs rich thrombus (Case 2), showing : **a**, **b -** a gradual increase in porosity towards the periphery and an outer layer of fibrin; **c**-to-**f** : the compact core formed of polyhedrocites.

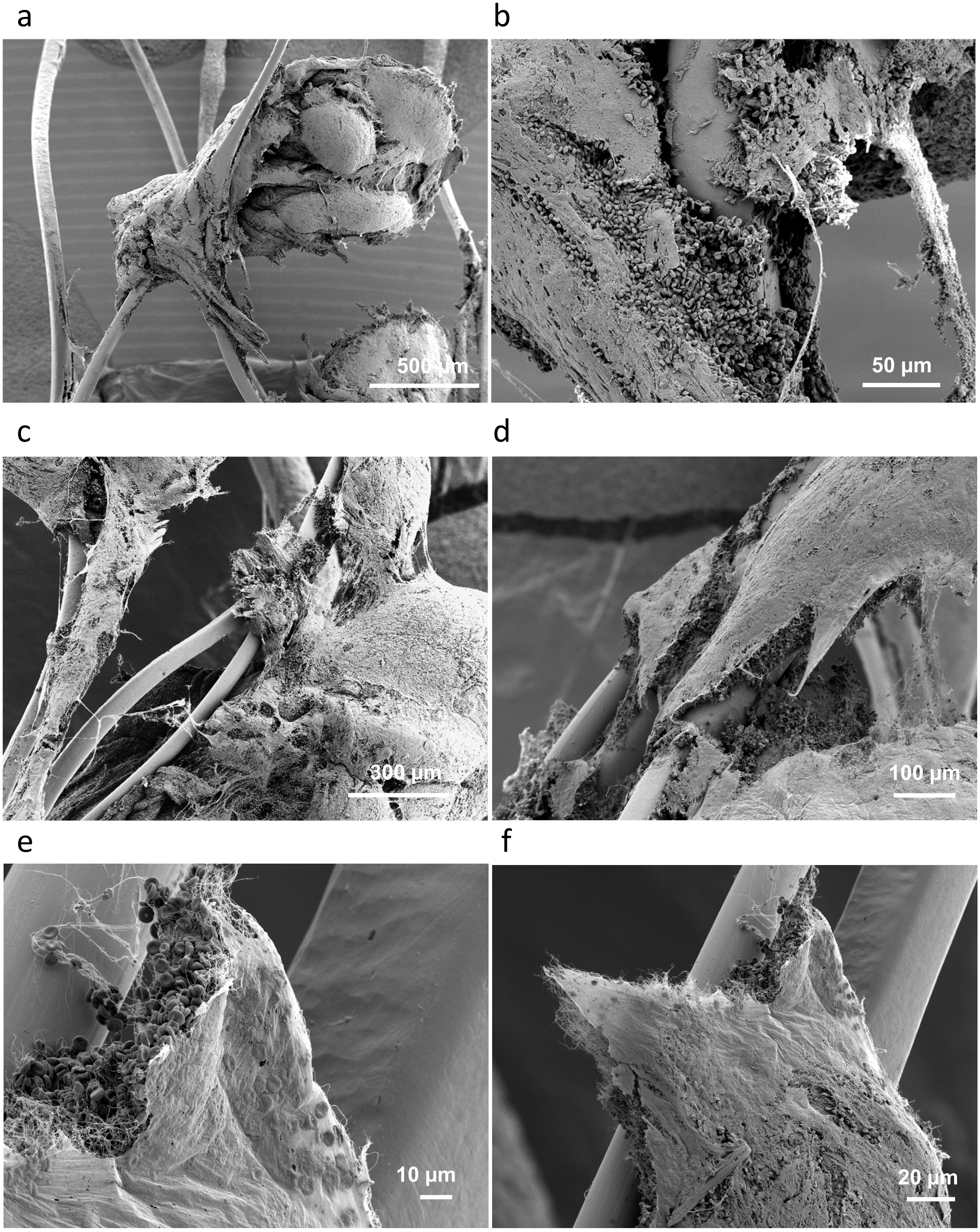

Supplementary Fig. S2 : Stent protruding through the RBCs rich thrombus (Case 2), in regions of loose cellular packing.

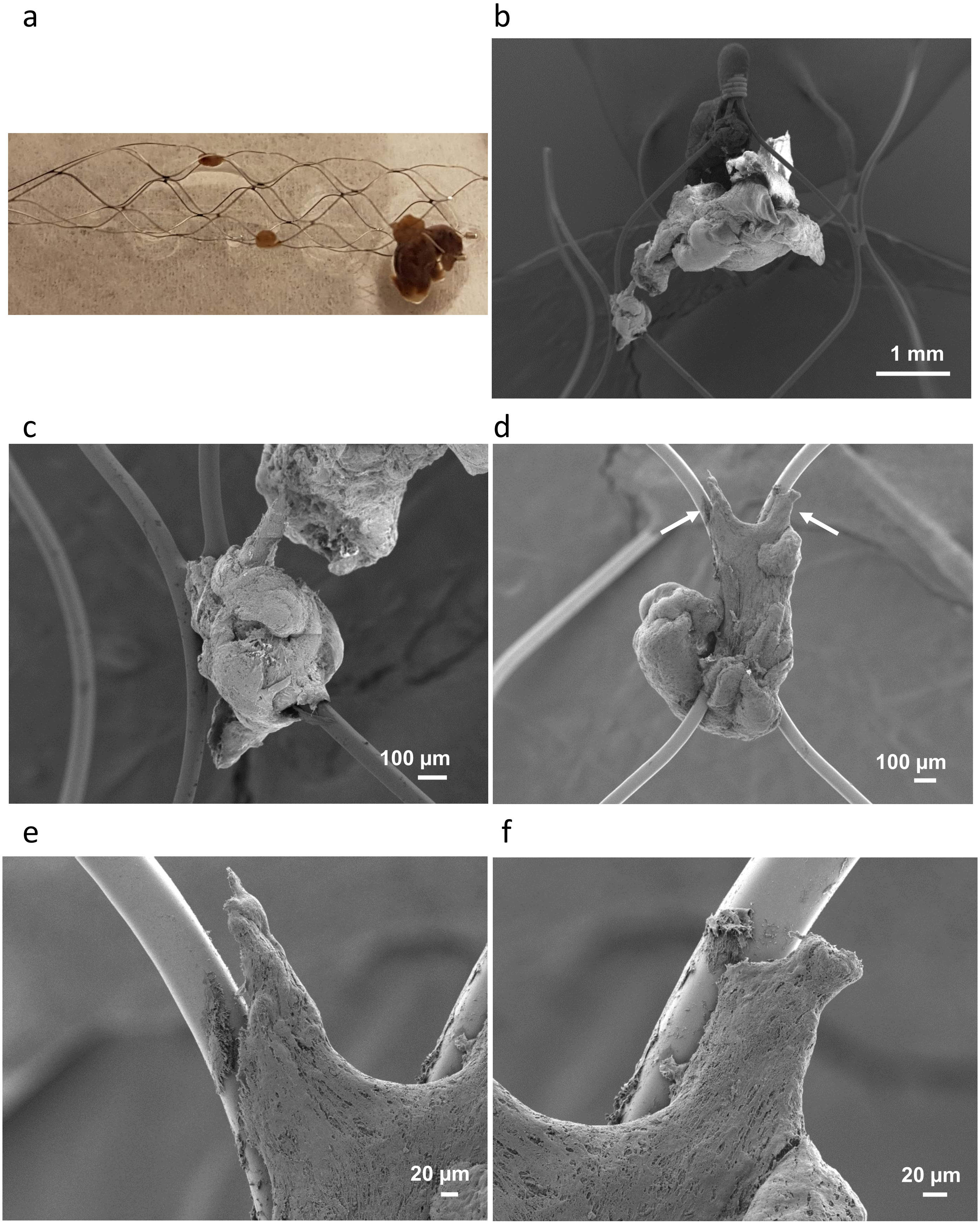

Supplementary Fig. S3 : Intermediate thrombus incorporated into the stent (Case 5). **a**. Optical micrograph. **b**-to-**d** : SEM of thrombus wrapped around the stent. **d**-to-**f** : strings of fibrin wetting the stent surface (indicated by arrows).

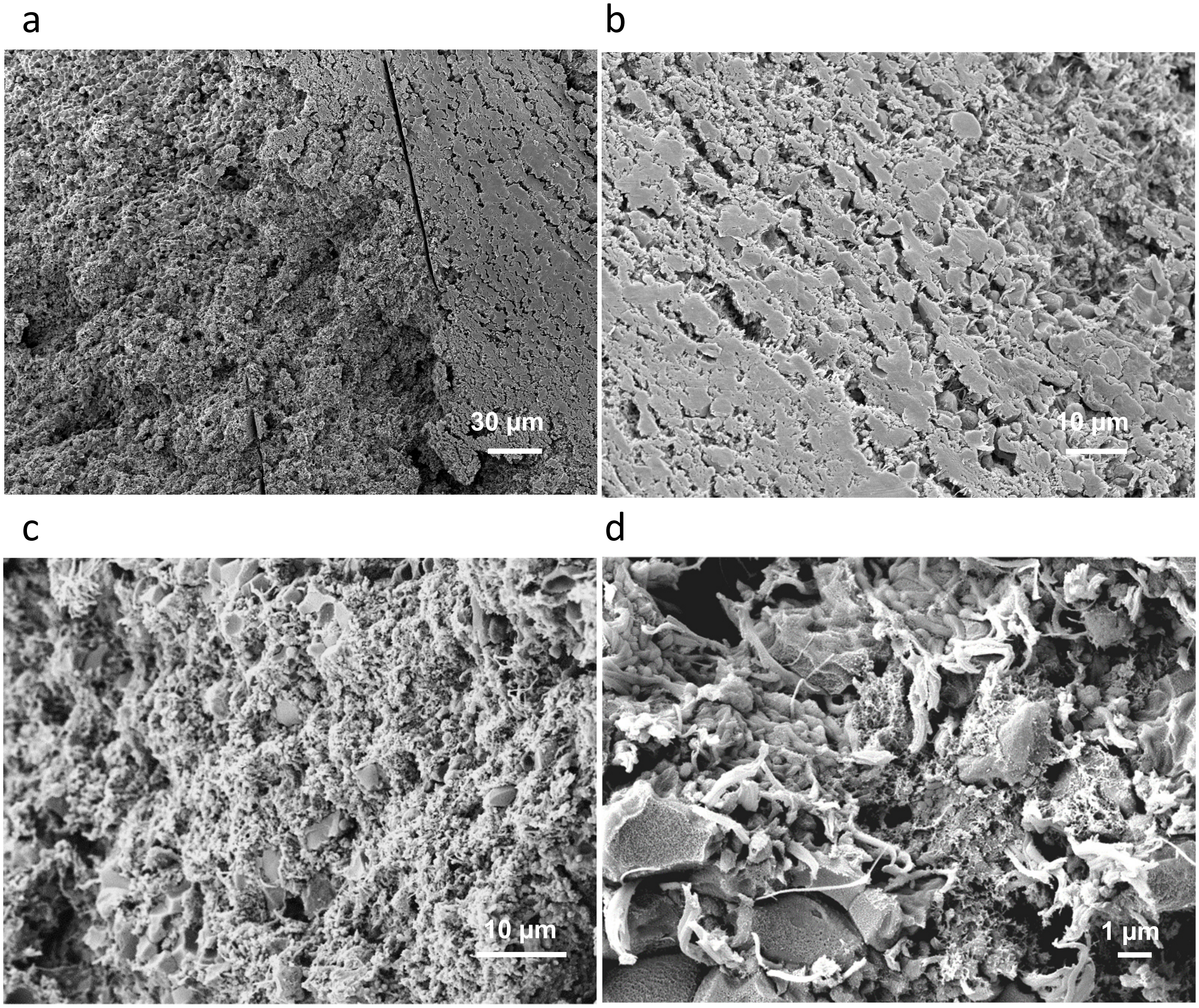

Supplementary Fig. S4 : Cross sections of an intermediate thrombus, at various magnifications, showing a compact core with RBCs encased in a fibrin-platelet matrix.

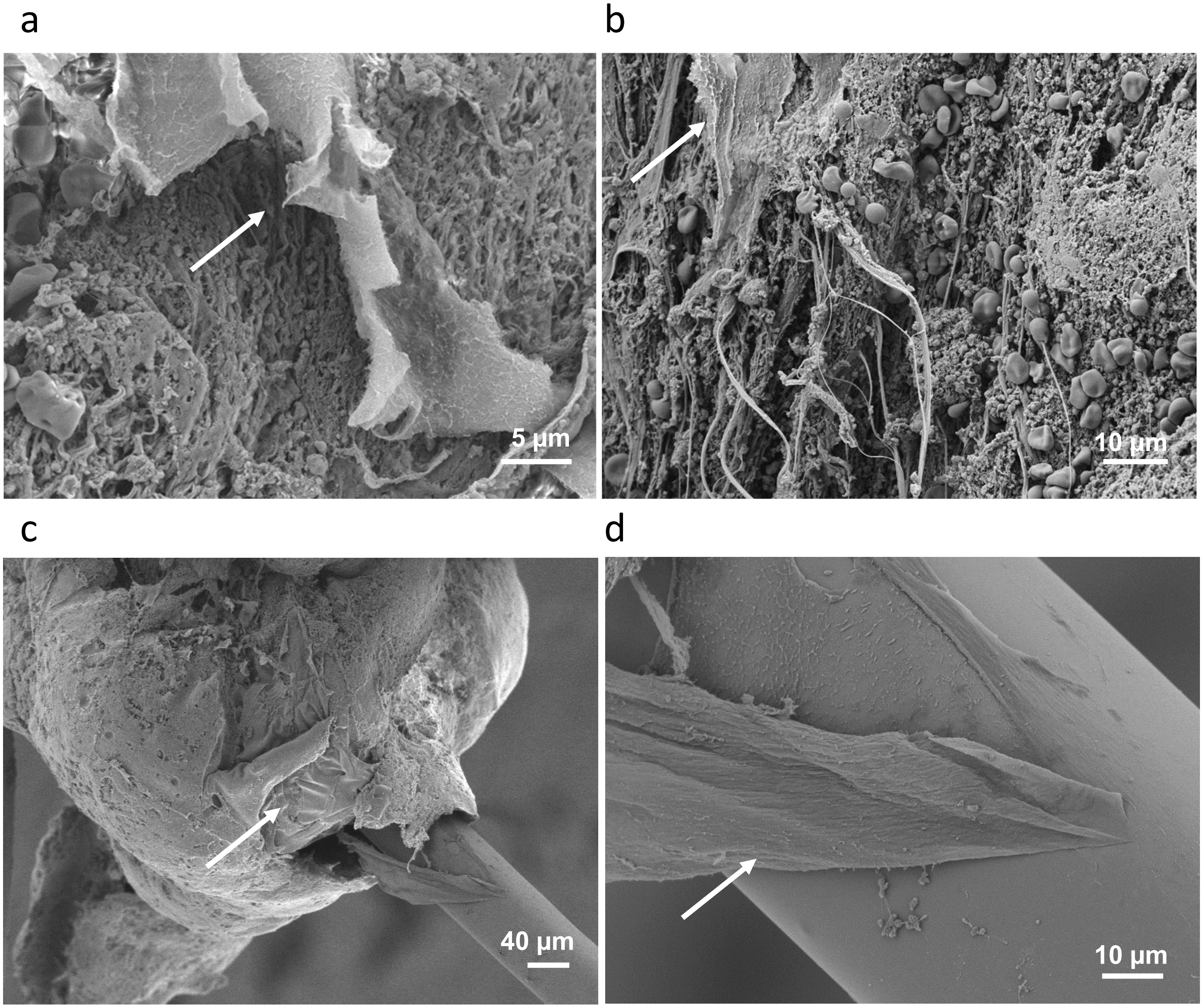

Supplementary Fig. S5 : Retrieved thrombus displays residual vascular tissue at its surface (as indicated by arrows).

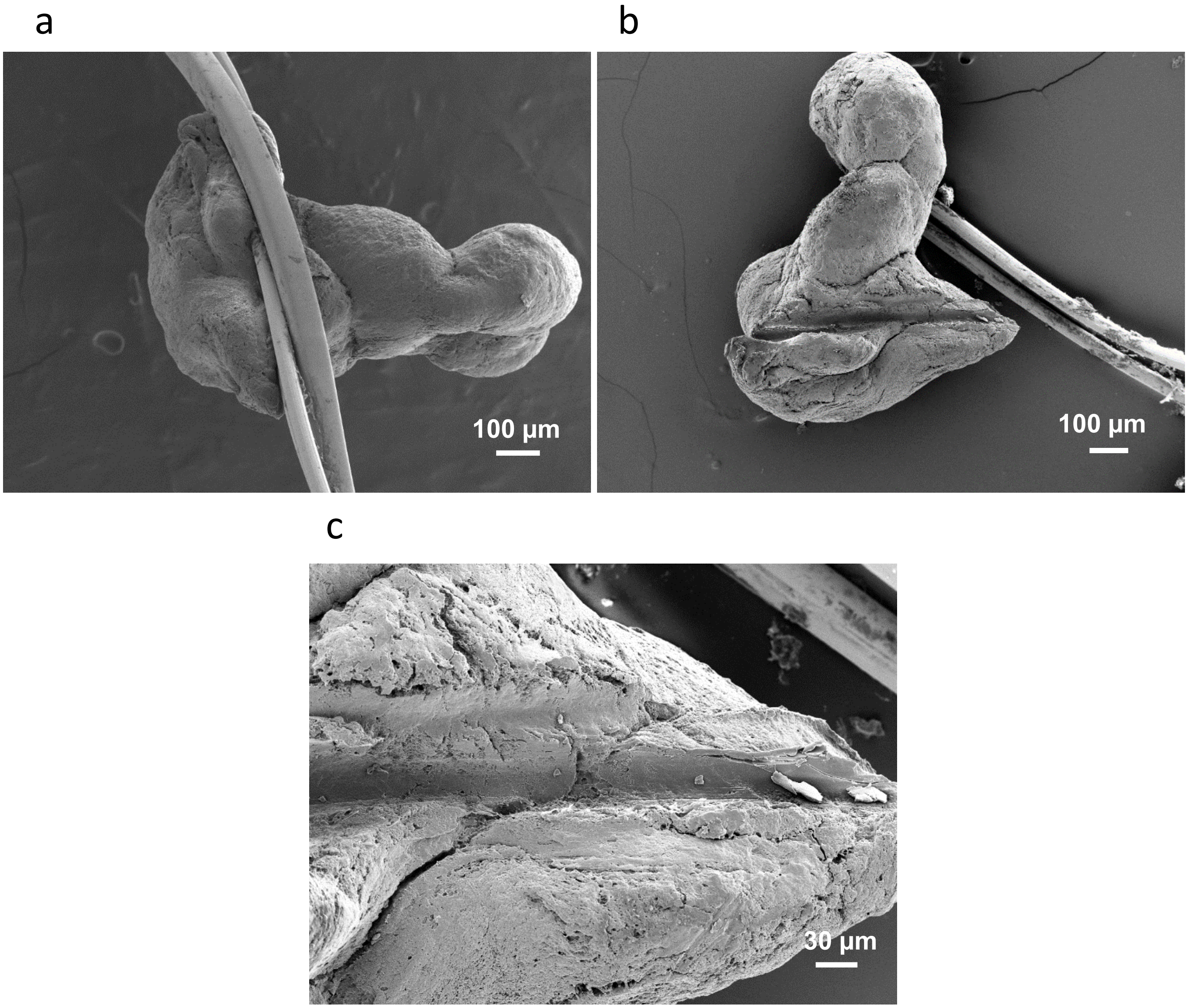

Supplementary Fig. S6 : A thrombus trapped between the stent struts (part of thrombus retrieved in Case 7). **a**. Thrombus trapped following MTB. **b, c** – thrombus was released from the struts for SEM observation, as such that its contact surface with the struts becomes conspicuous.
